# Role of the 2 zebrafish *survivin *genes in vasculo-angiogenesis, neurogenesis, cardiogenesis and hematopoiesis

**DOI:** 10.1186/1471-213X-9-25

**Published:** 2009-03-26

**Authors:** Mieke Delvaeye, Astrid De Vriese, Femke Zwerts, Inge Betz, Michael Moons, Monica Autiero, Edward M Conway

**Affiliations:** 1 KU Leuven, VIB Vesalius Research Center (VRC), Gasthuisberg O&N-1, Herestraat 49, 3000 Leuven, Belgium

## Abstract

**Background:**

Normal growth and development of organisms requires maintenance of a dynamic balance between systems that promote cell survival and those that induce apoptosis. The molecular mechanisms that regulate these processes remain poorly understood, and thus further *in vivo *study is required. Survivin is a member of the inhibitor of apoptosis protein (IAP) family, that uniquely also promotes mitosis and cell proliferation. Postnatally, survivin is hardly detected in most tissues, but is upregulated in all cancers, and as such, is a potential therapeutic target. Prenatally, survivin is also highly expressed in several tissues. Fully delineating the properties of survivin *in vivo *in mice has been confounded by early lethal phenotypes following *survivin *gene inactivation.

**Results:**

To gain further insights into the properties of survivin, we used the zebrafish model. There are 2 zebrafish *survivin *genes (*Birc5a *and *Birc5b*) with overlapping expression patterns during early development, prominently in neural and vascular structures. Morpholino-induced depletion of *Birc5a *causes profound neuro-developmental, hematopoietic, cardiogenic, vasculogenic and angiogenic defects. Similar abnormalities, all less severe except for hematopoiesis, were evident with suppression of *Birc5b*. The phenotypes induced by morpholino knockdown of one *survivin *gene, were rescued by overexpression of the other, indicating that the *Birc5 *paralogs may compensate for each. The potent vascular endothelial growth factor (VEGF) also entirely rescues the phenotypes induced by depletion of either *Birc5a *and *Birc5b*, highlighting its multi-functional properties, as well as the power of the model in characterizing the activities of growth factors.

**Conclusion:**

Overall, with the zebrafish model, we identify survivin as a key regulator of neurogenesis, vasculo-angiogenesis, hematopoiesis and cardiogenesis. These properties of survivin, which are consistent with those identified in mice, indicate that its functions are highly conserved across species, and point to the value of the zebrafish model in understanding the role of this IAP in the pathogenesis of human disease, and for exploring its potential as a therapeutic target.

## Background

For normal homeostasis in multicellular organisms, there exists a delicate balance between cell proliferation and cell death, maintenance of which is required to prevent pathological outcomes including developmental abnormalities, cancer, autoimmune diseases, degenerative disorders and poor outcome following wounds and ischemic injury. The major physiologic means by which cell death is achieved in an organism is via apoptosis, a tightly regulated and highly conserved process. In spite of major gains in characterizing the apoptotic pathways *in vitro*, a better understanding of the precise cellular mechanisms of apoptosis in different tissues and developmental time points *in vivo *is still required.

The inhibitor of apoptosis proteins (IAPs) are a family of conserved caspase inhibitors originally identified in baculoviruses as proteins capable of preventing virus-mediated cell death in insect cells. Survivin is the smallest of the human inhibitor of apoptosis proteins (IAPs), and has several unique features (reviewed in [1]). It possesses one baculovirus IAP repeat (BIR) domain that is essential for caspase interference, and contains a C-terminal alpha-helical coiled-coil domain [2] that is important for mitosis. Survivin functions as a chromosome passenger protein, complexing with aurora B, borealin and INCENP [3]. Survivin also promotes cell cycle progression [4], and is highly expressed by proliferating cells, while being barely detectable in quiescent adult tissues [5]. Its uniformly elevated expression in essentially all cancers has rendered survivin a therapeutic target (reviewed in [6,7]), and thus a critical understanding of the properties of survivin is key for its successful introduction into the clinic.

In both humans and mice, there is a single *survivin *gene that generates several different protein products due to alternative pre-mRNA splicing (reviewed in [8]). Transgenic mouse studies revealed that the major, full-length form of survivin is crucial for normal leukocyte [9] and hepatocyte function [10], hematopoiesis [11], and optimal angiogenic response to injury [2,8,12]. Elucidating its developmental role has been limited by the fact that *survivin *gene inactivation in mice causes early embryonic lethality [3,10]. Nonetheless, conditional gene inactivation studies indicate that survivin is essential for brain development [13], angiogenesis and cardiogenesis [14]. In *Xenopus laevis*, there are 2 *survivin *genes, overexpression of one which induces endothelial proliferation [15], while augmented expression of the other had inexplicably lethal effects. *Danio rerio *(zebrafish) also have 2 *survivin *genes. Ma et al [16] reported that survivin is important in angiogenesis during zebrafish development, but these studies were limited, as the authors did not examine early developmental time points, they restricted their analyses to only one of the genes, and they did not evaluate the importance of survivin in other organ systems.

To gain further insights into the developmental role of survivin, we used the zebrafish model and detailed the spatio-temporal expression patterns of the 2 *survivin *genes and characterized their functions. Zebrafish *survivin 1 *(*Birc5a*) and *survivin 2 *(*Birc5b*) both prevent apoptosis and promote cell proliferation. While they have overlapping patterns of distribution and function, *Birc5a *predominates in most systems. Consistent with its role in the mouse, both zebrafish *survivin *genes play critical roles in regulating developmental vasculogenesis, angiogenesis, neurogenesis, cardiogenesis, valvulogenesis and hematopoiesis. The model highlights the conservation of survivin's functions across species, and points to the relevance of utilizing the zebrafish model to evaluate its mechanisms of action, findings that may be extrapolated to human disease.

## Results

### Orthologues of Survivin in zebrafish

A BLAST search for orthologues of human *survivin *in zebrafish revealed 2 *survivin *genes (*Birc5a *and *Birc5b*) located on chromosomes 12 and 23, respectively. Using the CLUSTAL W program, the putative predicted proteins were aligned with the orthologous proteins found in human, mouse, *Xenopus laevis *and *Xenopus tropicalis *(Additional file 1A). Human survivin is 142 amino acids long, comprised of a 15 amino acid N-terminal domain, a BIR domain (amino acids 16–87), and a C-terminal coiled-coil domain. The proteins encoded by *Birc5a *and *Birc5b *exhibit 45–55% overall similarity with those from other species and with each other (Additional file 1B). The greatest sequence conservation between Birc5a and Birc5b resides in the BIR domain (79% similarity). From computer analysis (COILS: ), the C-terminal domains of the zebrafish survivin proteins do not form coiled-coil alpha-helical structures, a region that in murine survivin, interacts with the mitotic spindle.

### Expression patterns of *Birc5a *and *Birc5b*

**Figure 1:**
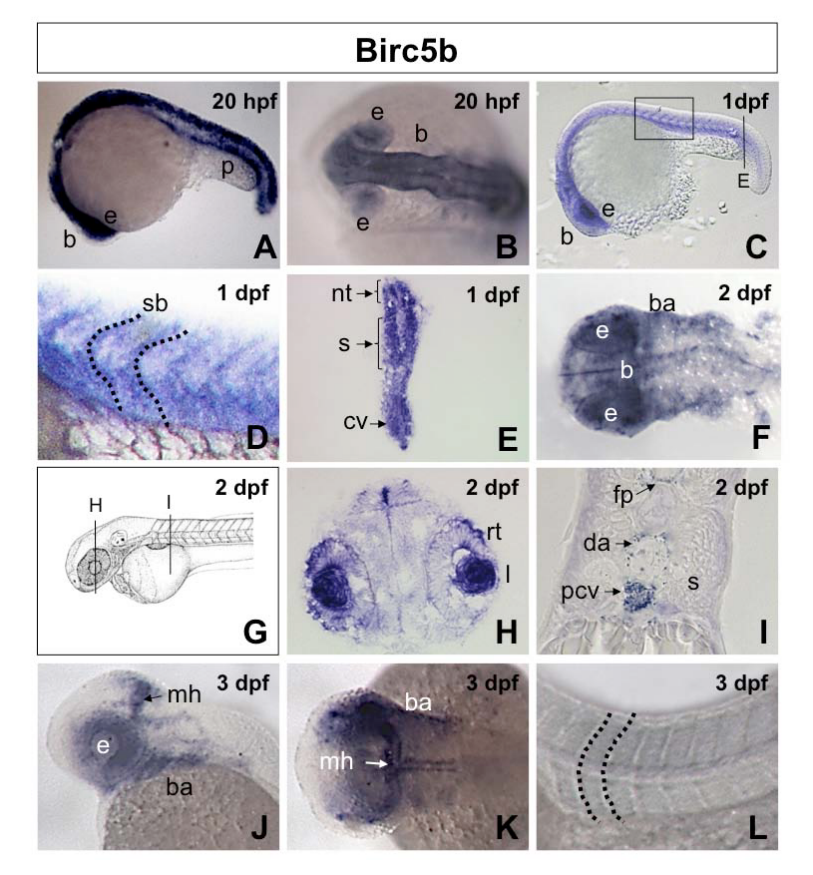
***Birc5b *spatiotemporal expression in zebrafish embryos**. **A, B**. Lateral (A) and dorsal (B) views of 20 hpf (20 somites) embryos revealing expression of *Birc5b *in the neural tube, brain, pronephric duct and eyes. **C**. Lateral view 1 dpf (30 somites) embryo, showing *Birc5b *expression in brain, eye, neural tube, somite and intersomite boundaries, with higher magnification in **D**. **E**. Transverse section through 1 dpf embryo (from C), revealing expression of *Birc5b *in neural tube, somites and caudal vein plexus. **F**. Dorsal view of head region of 2 dpf embryo. *Birc5a *detected in brain, floor plate and branchial arches. **G**. Diagram of 2 dpf embryo with transverse sections in panels **H **and **I**. Transverse sections through 2 dpf embryo reveals expression in retina and iris (H), floor plate, dorsal aorta, posterior cardinal vein; not in somite (I). **J-L**. Lateral (J) and dorsal (K, L) views of 3 dpf embryo; expression of *Birc5b *at midbrain-hindbrain barrier, branchial arches and eyes; not in region of axial vessels, somites or intersomite boundaries (L). nt: neural tube, p: pronephric duct, b: brain, e: eye, rt: retina, I: iris, mh: midbrain-hindbrain barrier, fp: floor plate, ba: branchial arches, s: somite, sb: intersomite boundary, da: dorsal aorta, pcv: posterior cardinal vein, cv: caudal vein plexus.

Ma et al [16] previously described expression of *Birc5a *in 26 hpf zebrafish embryos restricted to the developing brain, neural tube, and in cells surrounding the axial vessels. We performed detailed studies of the expression patterns of both *Birc5a *and *Birc5b *by *in situ *hybridization using gene-specific probes (Additional file 2). The 2 genes entirely overlap in their spatio-temporal patterns of expression. Since there are no prior reports on the expression of *Birc5b *in the zebrafish embryo, we therefore only provide images for *Birc5b *(Figure 1). *Birc5a *and *Birc5b *were detected as maternal messages throughout the embryo 1 hour post fertilization (hpf) (not shown). Similar to the report by Ma et al [16] for *Birc5a*, by 20–24 hpf (20–30 somites), expression of both genes was prominent in neural tissues, including the entire brain, neural tube, the floor plate, and the midbrain-hindbrain barrier (Figure 1A, B). In contrast to the report of Ma et al, at 1 dpf, we also found *Birc5 *expression in the ventral somites, at the somite boundaries, and in the caudal vein plexus, and in the eye (Figure 1B–E). Although evident from 20 hpf, at 2 dpf, there was more prominent expression of both *Birc5 *transcripts in the lens and retina of the eyes, in the major axial vessels, and in the branchial arch primordia (Figure 1F–I). Notably, by 2 dpf, expression in the somites was almost absent (Figure 1I). By 3 dpf, both *Birc5 *transcripts were detected in all the branchial arches, in brain, at the midbrain-hindbrain boundary, in the eyes, but was almost entirely absent in the axial vessels and somites (Figure 1J–L). Overall, the expression patterns of the two zebrafish *survivin *genes are indistinguishable during development, and are predominantly localized to neural, vascular and ocular structures, with transient expression in the somites and axial vessels during their formation.

### Zebrafish *Birc5 *morpholino knockdowns

To assess the functions of *Birc5a *and *Birc5b*, we used morpholinos to knock down each of the genes. Morpholinos were selected to target sites within each orthologue with the most sequence divergence, including the start codon (ATG morpholinos). Specificity of ATG morpholinos was confirmed by also using morpholinos that target pre-mRNA splice sites or 5' UTR regions (Additional file 3). Controls were performed by injecting a mismatch morpholino (GeneTools, Philomath, Oregon, USA). Each morpholino dose was tested on at least 100 embryos. All morpholinos (ATG, splice site and UTR) for one gene gave identical phenotypes; the results reported reflect those with ATG-morpholinos. We additionally confirmed gene-specificity by *in vitro *transcription-translation studies. Even at high doses, *Birc5a *morpholinos had no effect on *Birc5b *expression, and similarly, *Birc5b *morpholinos did not affect *Birc5a *expression (Additional file 4[17,18]). Finally, in all studies, we excluded p53-mediated off-target effects on apoptosis and the observed *survivin *morpholino-induced phenotypes, by co-injecting p53 morpholinos, as described [19] (data not shown).

### *Birc5 *in neural development

**Figure 2:**
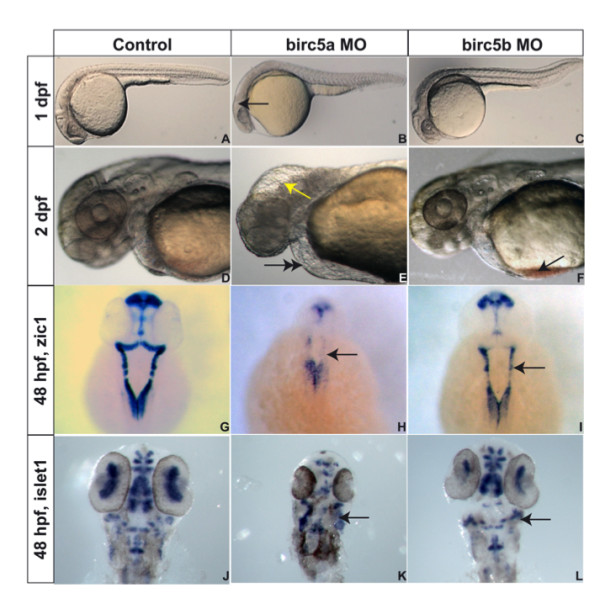
***Birc5 *in neurodevelopment**. Brightfield microscopy of AB zebrafish embryos. A-F: Embryos oriented with head to left. Lateral views at 1 dpf (A, B, C) and 2 dpf (D, E, F), the latter being higher power views of head region. Depletion of *Birc5a *results in lack of brain development, revealed at 1 dpf (B, arrow) and 2 dpf (E, arrow), with fluid in 4^th ^ventricle, compared to controls (A, D). At 2 dpf, *Birc5a *knockdown causes cardiogenic defects and pericardial edema (E, double arrow), not observed in controls (D). Majority of *Birc5b *depleted embryos do not exhibit phenotypic abnormalities under brightfield microscopy at 1 dpf (C) compared to controls (A). At 2 dpf, *Birc5b *knockdown embryos have smaller head and brain, and accumulate blood in the sinus venosus (F, arrow). *Zic1 *expression to detect neural tissue, is decreased by *Birc5a *depletion (H, arrow), compared to controls (G). A similar but less dramatic diminution of *Zic1 *expression is observed with morpholino knockdown of *Birc5b *(I, arrow). Depletion of *Birc5a *also induces disorganization of motor neurons, detected by expression of *islet1 *(K, arrow), compared to controls (J). *Birc5b *morphants exhibit less severe but still evident, suppression of *islet1 *expression (L, arrow).

*Birc5a *and *Birc5b *morpholino-injected wild-type AB embryos were assessed by brightfield microscopy (Figure 2). After injection of 0.5 ng *Birc5a *morpholino, morphant embryos at 1 dpf (Fig 2A, B) and 2 dpf (Fig 2D, E) displayed microcephaly (42%, n = 162 at 1 dpf; 57%, n = 157 at 2 dpf) with fluid accumulation in the 4^th ^ventricle (Fig 2E). The effects of *Birc5b *depletion on brain development using the highest morpholino dose were less dramatic than with the *Birc5a *knockdown, with 10–15% of *Birc5b *morphants exhibiting microcephaly at 1 dpf (n = 143) and 2 dpf (n = 105) (Figure 2C, F).

Disturbances in neuronal development were, however, more readily detectable in both *Birc5a and Birc5b *mutants by *in situ *hybridization with probes to detect *Zic1*, a pan-neural marker [20] (Figure 2G–I), and *Islet1*, a marker of primary motor neurons [21] (Figure 2J–L). *Zic1 *staining of the brain was decreased in the 2 dpf *Birc5a *morphants as compared to controls, reflecting the almost total absence of neural cells. The reduction was also evident in the *Birc5b *morphants, to a lesser extent when compared to the *Birc5a *morphant. Similarly, *islet1 *staining of the *Birc5a *morphants revealed disorganized or absent motor neurons, while the *Birc5b *morphants were also affected, but again, less severely.

Overall, both *Birc5 *genes are critical for normal neural development, with *Birc5a *predominating.

### *Birc5 *in vasculogenesis and angiogenesis

**Figure 3:**
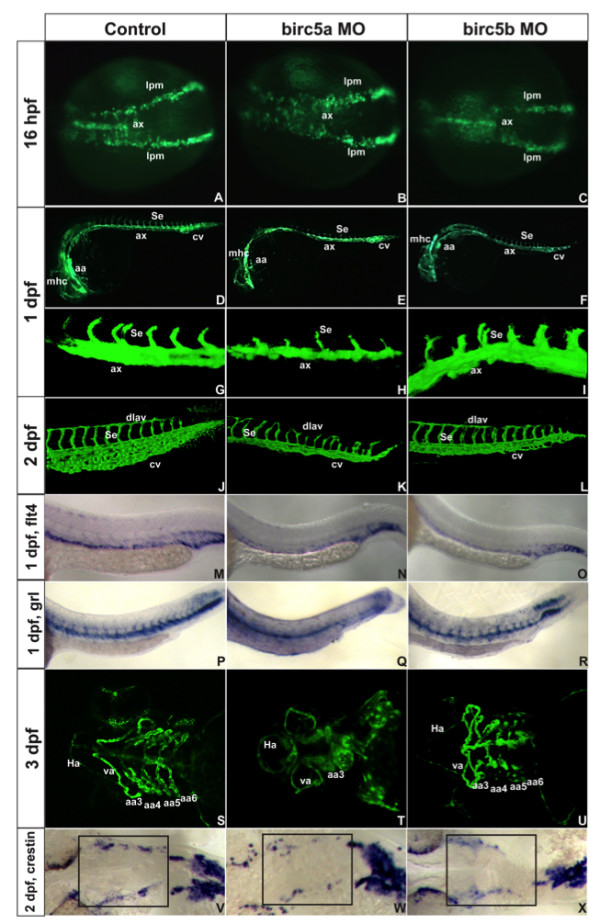
***Birc5 *in vasculogenesis and angiogenesis**. *Tg*(*Fli*:eGFP) (A-L) and *Tg*(*Flk1*:GFP) (S-U) embryos. A-C: 16 hpf (14 somites), *Birc5a *morphants with angioblast migration defects from lateral plate mesoderm (B) which are minor in *Birc5b *morphants (C). D-F: 1 dpf, *Birc5a *(E, H) and *Birc5b *(F, I) morphants have thinner axial vessels and poor caudal vein plexus development. G-I: 1 dpf *Birc5a *depletion (H) delays intersomitic vessels; not with *Birc5b *depletion (I). J-L: 2dpf *Birc5a *morphants with abnormal dorsal longitudinal anastomic and intersomitic vessels. Both morphants have poorly developed caudal vein plexus. M-O: *flt4 *at 1 dpf is reduced in posterior cardinal vein in both morphants (N, O). P-R: *gridlock *(*grl*) at 1 dpf is reduced with *Birc5a *depletion (Q), but not with *Birc5b *(R). S-U: 3 dpf *Birc5a *morphants have hypoplastic aortic arches (T). *Birc5b *depletion at 3 dpf causes hypoplasia of aortic arches 5–6 (U). V-X: *Birc5a *depletion decreases neural crest cells that migrate to branchial arches, detected with *crestin *probe. *Birc5b *depletion (L) reduces neural crest cells. ax: axial vessels, mhc: midbrain-hindbrain channel, Se: intersomitic vessels, aa: aortic arch, cv: caudal vein plexus, pcv: posterior cardinal vein, da: dorsal aorta, dlav, dorsal longitudinal anastomic vessel, Ha: hypobranchial artery, va: ventral aorta, lpm: lateral plate mesoderm.

Recently, Ma et al [16] reported that *Birc5a *knockdowns primarily induce angiogenic abnormalities, but without affecting vasculogenesis. We extended on their work by examining vasculo-angiogenesis in *Birc5a *and *Birc5b *knockdown embryos at different developmental time points, using *Tg(Fli:eGFP) or Tg(Flk1:GFP) *embryos (Zebrafish International Resource Center [22]) which express green fluorescent protein (GFP) in endothelial cells (Figure 3).

Precursor angioblasts in zebrafish arise at 12 hpf (6 somites) in the lateral plate mesoderm and migrate from 14 hpf (10 somites) toward the midline, where they coalesce to form primary axial vessels [22]. We first established that depletion of *Birc5a *or *Birc5b *does not induce a defect in mesodermal development, by determining that expression of the mesodermal marker, *no tail *(*ntl*) [23] in both morphants at 6 hpf, as compared to control zebrafish embryos, was not different (not shown).

We then studied the effect of depleting *Birc5a *and *Birc5b *with the highest morpholino doses (2 ng and 4 ng, respectively) on vasculogenesis and angiogenesis. At 16 hpf (14 somites), *Birc5a *morphant angioblasts migrated in a disorganized fashion, at different rates, with some remaining at the lateral plate mesoderm (Figure 3A, B). Angioblasts in *Birc5b *morphants migrated normally to the midline at 16 hpf (Figure 3C), but there was reduced signal intensity and thickness of the coalescing axial vessels (22% of embryos, n = 147). With depletion of *Birc5a*, the dorsal aorta and posterior cardinal vein at 1 dpf and 2 dpf were thinner (45–52%), with a smaller caudal vein plexus (45–52%) (Figure 3D, E, G, H, J, K). A similar effect, although not as prominent and not involving the posterior cardinal vein, was also evident in *Birc5b *morphants (Figure 3F, I, L). The findings were better visualized following *in situ *hybridization of 1 dpf embryos with probes *flt4 *and *gridlock *(*grl*) that detect the posterior cardinal vein and the dorsal aorta/intersomitic vessels, respectively [24] (Figure 3M–R). The *Birc5a *morphants also exhibited delayed sprouting of intersomitic vessels (ISVs), which were occasionally absent, but otherwise were often thin, misdirected, lacking connections, and associated with interruption of the dorsal longitudinal anastomotic vessels. These findings, in concert with the fact that *Birc5 *expression was transiently detected at the somite boundaries, suggests a role for survivin in ISV patterning and vessel guidance (reviewed in [25]). Interestingly and in contrast, formation of the intersomitic vessels and dorsal longitudinal anastomotic vessels remained intact in the *Birc5b *morphants.

By 3 dpf, there was underdevelopment and irregular patterning of cranial blood vessels of both the *Birc5a *and *Birc5b *morphants (see Additional Files 5, 6, 7) as compared with controls. Only 1 of the branchial arches was seen in the majority of the *Birc5a *morphants (61%) (Figure 3S, T), while the 5^th ^and 6^th ^branchial arches in the *Birc5b *morphants were either absent or hypoplastic (65%) (n = 160) (Figure 3U). In attempting to explain the branchial arch defects, we performed *in situ *hybridization studies at 2 dpf with crestin, a post migratory neural crest cell marker [26]. In both the *Birc5a *and the *Birc5b *morphants, but more prominent with depletion of *Birc5a*, there were fewer crestin-positive neural crest cells in the region of the neural crest, corresponding to that which is critical for development of the branchial arches [27] (Figure 3V–X).

In summary, both *Birc5 *genes are important for normal vasculogenesis, angiogenesis, and vascular patterning. Although the 2 genes functionally overlap, again, *Birc5a *appears to play a more prominent role.

### *Birc5 *in hematopoiesis

**Figure 4:**
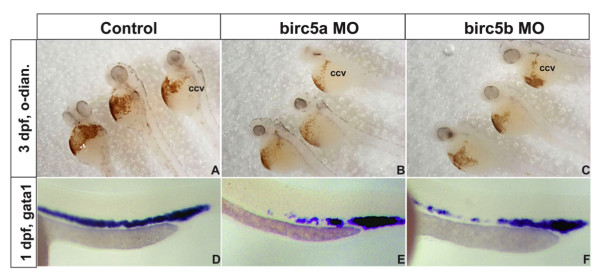
***Birc5 *in hematopoiesis**. A-C: Depletion of *Birc5a *(B) or *Birc5b *(D) causes a reduction in erythropoiesis, shown by staining of erthrocytes with o-dianisidine (control, A). D-F: The preceding is consistent with decreased expression of *gata1 *by *in situ *hybridization in both gene knockdowns (E, F) as compared to control (D). ccv: common cardinal vein or duct of Cuvier, o-dian.: o-dianisidine.

Since, in the zebrafish, at least a subset of hematopoietic and endothelial lineages arise from a common hemangioblast [28], we also examined *Birc5a *and *Birc5b *morphants for defective hematopoiesis. Erythrocytes in 3 dpf embryos were identified by staining for hemoglobin with *o*-dianisidine. Depletion of *Birc5a *or *Birc5b *resulted in a reduced number of erythrocytes in the ducts of Cuvier (Figure 4A–C). Moreover, *gata1 *expression, reflecting hematopoiesis at 1 dpf, was reduced in 35% of *Birc5a *morphants (n = 112), and 28% of *Birc5b *morphants (n = 115), as compared to controls (n = 70) (Figure 4D–F). Since defects in circulation may impact on the preceding findings, we examined hematopoiesis at 14 hpf, prior to development of a functional circulation. Flow cytometry of cellular suspensions of dechorianated *Tg(gata1:GFP*) embryos was used to quantify the number of Gata1+ cells relative to the total number of cells [29]. Depletion of either *Birc5a *or *Birc5b*, as compared to control injections, resulted in a decrease in the absolute number of Gata1+ cells from 3.37% for controls to 0.72% for *Birc5a*, and to 0.42% for *Birc5b*. In line with these findings, expression of hematopoietic genes *gata1, scl *and *Imo2 *[29], quantified by real-time PCR at 14 hpf and 18 hpf in AB zebrafish embryos, was significantly reduced (p < 0.01) by depletion of either *Birc5a *or *Birc5b*. Overall, the data support a role for both *Birc5 *genes in promoting hematopoiesis in the developing zebrafish embryo.

### *Birc5 *in cardiogenesis

**Figure 5:**
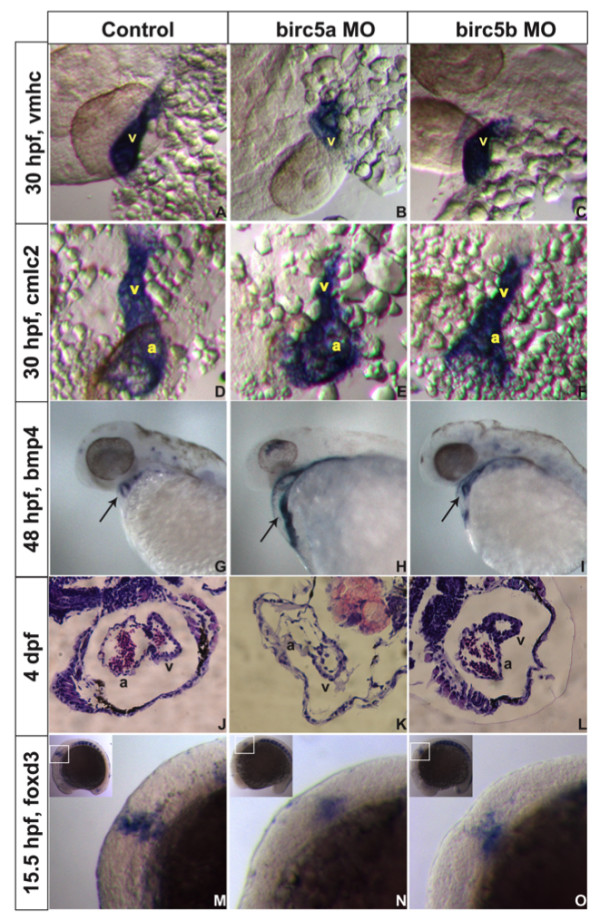
***Birc5 *in cardiogenesis**. *In situ *hybridizations (A-I, M-O) and histologic sections (J-L) on AB embryos. A-C: At 30 hpf, expression of cardiac ventricle marker *vmhc *is reduced with *Birc5a *depletion (A, B), and to lesser extent in *Birc5b *morphants (C). D-F: *cmlc2 *staining shows *Birc5a *(E) or *Birc5b *(F) morphants with impaired development of atrium and ventricle, compared to controls (D). G-I: At 48 hpf, *bmp4 *normally localizes in heart to reveal a ring-like structure, representing endocardial cushions of the atrio-ventricular valve (G, arrow). With depletion of *Birc5a*, *bmp4 *staining remains diffuse and ring structure is absent (H, arrow). In *Birc5b*-morphants, *bmp4 *localizes normally (I, arrow), but the ring is smaller. J-L: H&E stained histologic sections of hearts of normal embryos (J), and those depleted of *Birc5a *(K) and *Birc5b *(L) at 4 dpf. *Birc5a *morphants have thin-walled heart chambers, and little evidence of a-v valve formation. *Birc5b*-depleted embryos have smaller ventricles. M-O: Premigratory cardiac neural crest cells contributing to heart development, were detected by staining embryos at 15.5 hpf (13 somites) with *foxd3*. Compared to controls (M), premigratory neural crest cells were barely detectable in embryos depleted of *Birc5a *(N), and reduced in *Birc5b *morphants (O). a:atrium, v:ventricle.

Depletion of either *Birc5a *or *Birc5b *resulted in cardiovascular defects. At the highest morpholino dose, one third of *Birc5a *morphants displayed pericardial edema, while blood flow in the dorsal aorta and posterior cardinal vein was slowed or absent in at least half the embryos. The heart rate in both *Birc5a *and *Birc5b *morphants was significantly reduced (60 ± 8, 56 ± 8, and 86 ± 7 beats/minute in *Birc5a *morphants, *Birc5b *morphants, and controls, respectively; p < 0.001 *versus *controls). *In situ *hybridization at 30 hpf with the ventricle and cardiac-specific markers, ventricle myosin heavy chain (*vmhc*) and cardiac myosin light chain (*cmlc2*) [30], respectively, demonstrated that *Birc5a *morphant ventricles and hearts were smaller than controls (41%, n = 24 for vmhc; 42%, n = 76 for cmlc2) (Figure 5A, B, D, E). Although not as striking, *Birc5b *morphants also exhibited smaller ventricles and hearts at 30 hpf (Figure 5C, F) (26%, n = 31 for *vmhc*; 32%, n = 89 for *cmlc2*). At 48 hpf when atrio-ventricular (a-v) valve formation is underway, bone morphogenic protein 4 (*bmp4*) [31] transcripts progressively localize from the atrium and ventricle to myocardial cells at the valve-forming region [32]. With *Birc5a *depletion, redistribution of *bmp4 *did not occur in 38% (n = 62) of embryos (Figure 5G, H), predicting defects in valvulogenesis. This was evident in hematoxylin and eosin (H&E) stained sections of 4 dpf embryos, where there was little evidence of an a-v valve in the *Birc5a *morphants, at a time when the myocardial layer of the morphant ventricle was also thinner, as compared to control embryos (Figure 5J, K). In the *Birc5b *morphants, *bmp4 *became localized to the a-v valve, similar to controls, although the opening was smaller in 21% (n = 44) (Figure 5I). At 4 dpf, H&E stained sections also revealed smaller cardiac chambers (20%) (Figure 5L).

Although diminished vascular perfusion may contribute to cardiogenic defects [33], we hypothesized that an additional factor in the *Birc5 *morphants might be altered formation and/or migration of cardiac neural crest cells, a site of origin of cardiomyocytes in zebrafish [34]. Embryos at 15.5 hpf (13 somites) were therefore hybridized with the *foxd3 *probe to stain premigratory neural crest cells [35]. *Birc5a *depletion caused cardiac neural crest cell loss along the rostrocaudal axis (Figure 5M, N), particularly in the region critical for formation of the ventricle, a-v junction, and atrium (region referred to as "Group B" by Sato et al [36]). *Birc5b *depletion also resulted in loss of cardiac neural crest cells – again, not as dramatically as with *Birc5a *depletion (Figure 5O). Overall, the findings indicate that *Birc5a*- and *Birc5b*-dependent signals are important in maintaining the integrity of cardiac neural crest cells that in turn, contribute to normal cardiogenesis and valvulogenesis.

### *Birc5 *knockdowns result in increased apoptosis and decreased cell proliferation

Ma et al [16] reported that depletion of *Birc5a *caused increased apoptosis primarily in the neural tube and brain. Given the diverse pro-survival properties of survivin, we assessed the mechanisms of action of each zebrafish *survivin *gene in the neural and vascular systems.

**Figure 6:**
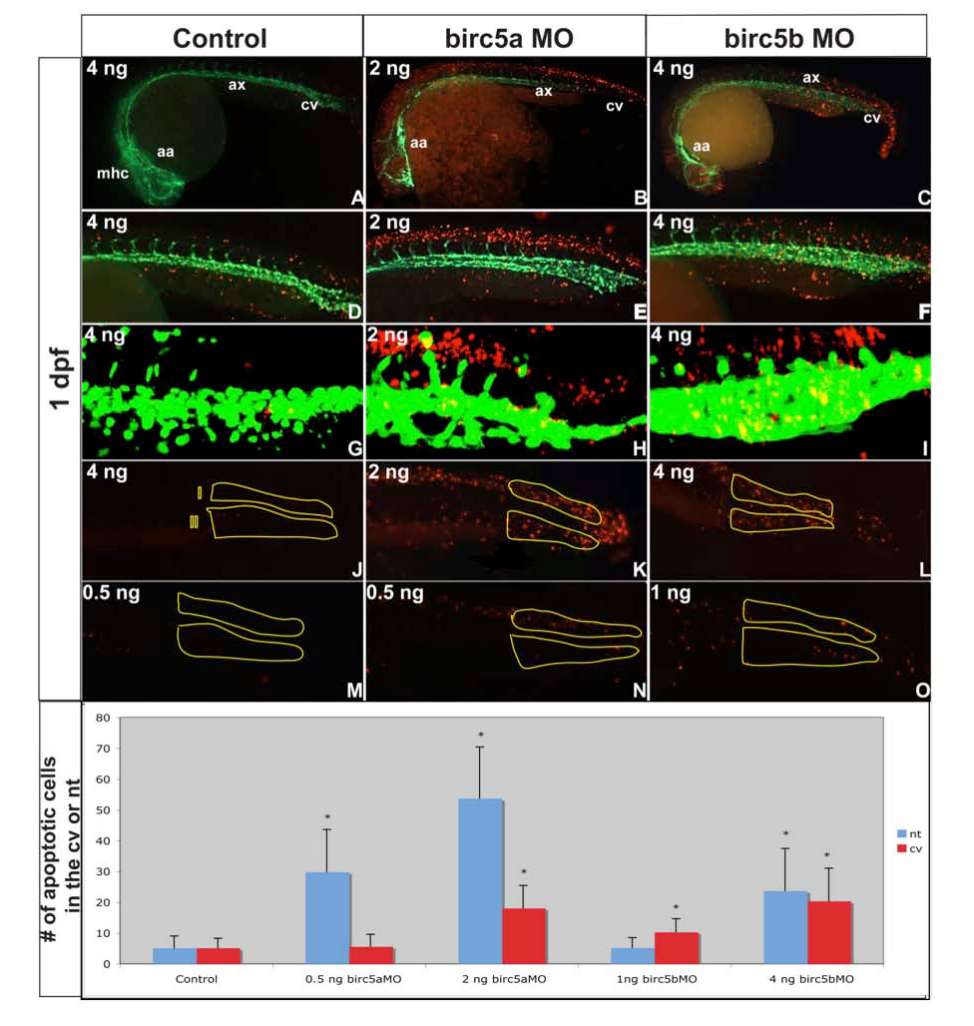
**Apoptosis in *Birc5*-depleted embryos**. Tg(*Fli*:eGFP) embryos reveal apoptosis (red) in relation to blood vessels (green) (A-O). A-I: Morphants at 1 dpf exhibit increased apoptosis, particularly in the brain (B) and along neural tube (E) in *Birc5a *morphants (morpholino dose 2 ng). *Birc5b *depletion with 4 ng of morpholino caused apoptosis in axial vessel region, caudal vein plexus, and neural structures (C, F). G-I: Confocal microscopy of 1 μm sagittal "slice" in region of caudal vein plexus and corresponding neural tube (excludes somites): Dose-dependent changes in apoptosis in caudal vein plexus region (J, region II) and corresponding neural tissue (J, region I) after *Birc5 *knockdowns was quantified at 1 dpf. High dose *Birc5a *morpholino (2 ng) or *Birc5b *morpholino (4 ng) causes significant increase in apoptosis in caudal vein plexus and neural tube (K, L). With lower *Birc5a *morpholino dose 0.5 ng), neural tube apoptosis remains significantly increased, but is almost absent in caudal vein plexus (N). Low dose *Birc5b *morpholino (1.0 ng) causes significant apoptosis in caudal vein plexus, but not in neural tube (O). Data presented in bottom panel. n = 30 × 3 independent experiments. * p < 0.05 relative to corresponding control. mhc: midbrain-hindbrain channel, aa: aortic arch, ax: axial vessels, cv: caudal vein plexus.

TUNEL staining of normal *Tg(Fli:eGFP) *embryos at 1 dpf (Figure 6, left column) and 2 dpf (not shown) revealed minimal evidence of apoptosis. In contrast, at a morpholino dose of 2 ng, *Birc5a *morphants exhibited significant apoptosis, mostly in the brain and spinal cord (Figure 6A, B, D, E), with lesser amounts in the region of the axial vessels and caudal vein plexus (Figure 6E, H). TUNEL staining in the *Birc5b *morphants at 1 dpf was, by comparison, less in the neural tissues, but more prominent in the region of the caudal vein plexus and axial vessels (Figure 6C, F). With both gene knockdowns, apoptosis remained spatially unchanged, but was increased at 2 dpf (not shown).

Quantification of TUNEL positive cells in the caudal vein plexus and the corresponding dorsal neural tube region confirmed that depletion of either *Birc5a *or *Birc5b *at the higher dose, caused a significant increase in the number of apoptotic cells in both regions (p < 0.05) (Figure 6J–L). However, when the *Birc5a *morpholino dose was decreased to 0.5 ng, apoptosis in the caudal vein plexus entirely resolved, while TUNEL staining in the neural tube persisted (Figure 6M, N), indicating a greater sensitivity of the neural structures to loss of *Birc5a*. Conversely, as the *Birc5b *morpholino dose was decreased, the effect on the neural tube diminished, while apoptosis in the region of the caudal vein plexus persisted (Figure 6M, O).

**Figure 7:**
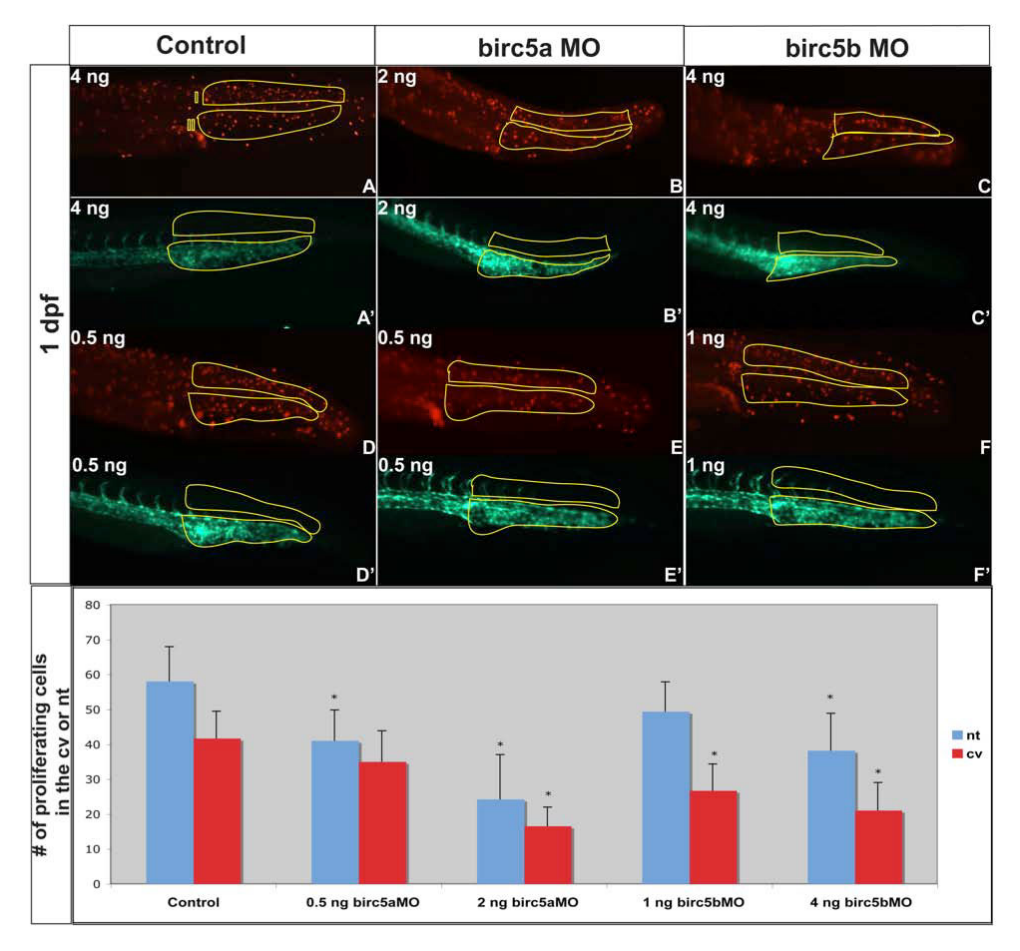
**Cell proliferation in *Brc5*-depleted embryos**. Tg(*Fli*:eGFP) embryos, were used to localize proliferating cells (red, A-F) in relation to blood vessels (green, A'-F') after *Birc5 *morpholino knockdowns. Proliferating cells immuno-detected by whole-mount staining of 2 dpf embryos with anti-phospho-Histone H3 antibodies (red). The number of proliferating cells in the caudal vein plexus and neural tube (see Figure 6J for regions) was quantified in embryos after high dose (B, C) or low dose (E, F) morpholino knockdowns. With high dose *Birc5a *or *Birc5b *morpholino (2 ng or 4 ng, respectively), there is a significant decrease in cell proliferation in the caudal vein plexus and the neural tube (B, C) as compared to the control (A). When the *Birc5a *morpholino knockdown dose is lowered (0.5 ng) (E), there is still a significant decrease in cell proliferation in the neural tube, but not in the caudal vein plexus, compared to control (D). Conversely, low dose *Birc5b *morpholino knockdown (1.0 ng) results in a significant diminution of cell proliferation in the caudal vein plexus, but not in the neural tube (F). Quantitative data are presented in the bottom panel. n = 30 × 3 independent experiments. * p < 0.05 relative to corresponding control.

We also quantified the effects of the two genes on cell proliferation (Figure 7). At 1 dpf, proliferating cells were present in the region of the caudal vein plexus and neural tube (Figure 7A, B). *Birc5a *depletion using 2 ng of morpholino interfered with cell proliferation in both regions, while the 0.5 ng dose (Figure 7D, E) only suppressed proliferation in the neural tube. Depletion of *Birc5b *also interfered with cell proliferation in the caudal vein plexus and neural tube, and this anti-proliferative effect in the neural tube disappeared at the lower morpholino dose (Figure 7C, F). Overall, the findings support the notion that both *Birc5 *genes interfere with apoptosis and promote cell proliferation during early zebrafish development, but that there are dose-dependent and site-specific distinguishing features.

#### Phenotype rescues of Birc5a and Birc5b morphants

We determined whether we could rescue the *Birc5 *morphants by co-injecting synthetic mRNAs encoding the open reading frame of either Birc5a or Birc5b. In a dose-dependent manner, co-injection of the respective *Birc5 *mRNA almost entirely rescued both the vascular and neural phenotypes induced by the highest morpholino dose (Table 1). Thus, at 2 dpf, the *Birc5a *morphants were rescued by co-injection of 1 ng of *Birc5a *mRNA, and the *Birc5b *morphants were rescued by 1 ng of *Birc5b *mRNA. Furthermore, co-injection of 1 ng of *Birc5b *mRNA could also partially rescue the *Birc5a *morphants, while *Birc5b *morphants could be completely rescued by *Birc5a *mRNA. The findings indicate that the 2 *survivin *genes may, under different conditions, compensate for each other.

**Table 1:** Rescue of neuro-vascular phenotypes with *Birc5 *mRNAs

		**Buffer or Specific mRNA Co-injected**				
		
	**Phenotype**	**Buffer**	***Birc5a *mRNA(n = 162)**	***Birc5b *mRNA(n = 180)**	***Birc5a *mRNA(n = 134)**	***Birc5b *mRNA(n = 173)**
***Birc5a *kd**	**Neuro**	61% (of 276)	8%	21%		
	
	**Vascular**	40% (of 276)	10%	22%		

***Birc5b *kd**	**Neuro**	26% (of 263)			5%	6%
	
	**Vascular**	38% (of 263)			4%	8%

*Birc5 *morpholinos were injected into *Tg(Fli:eGFP) *zebrafish embryos simultaneously with injections of specific mRNAs as shown, and phenotypic analyses were performed at 2 dpf. The incidence of neural or vascular abnormalities is illustrated.

#### Role of VEGF in regulating survivin

**Figure 8:**
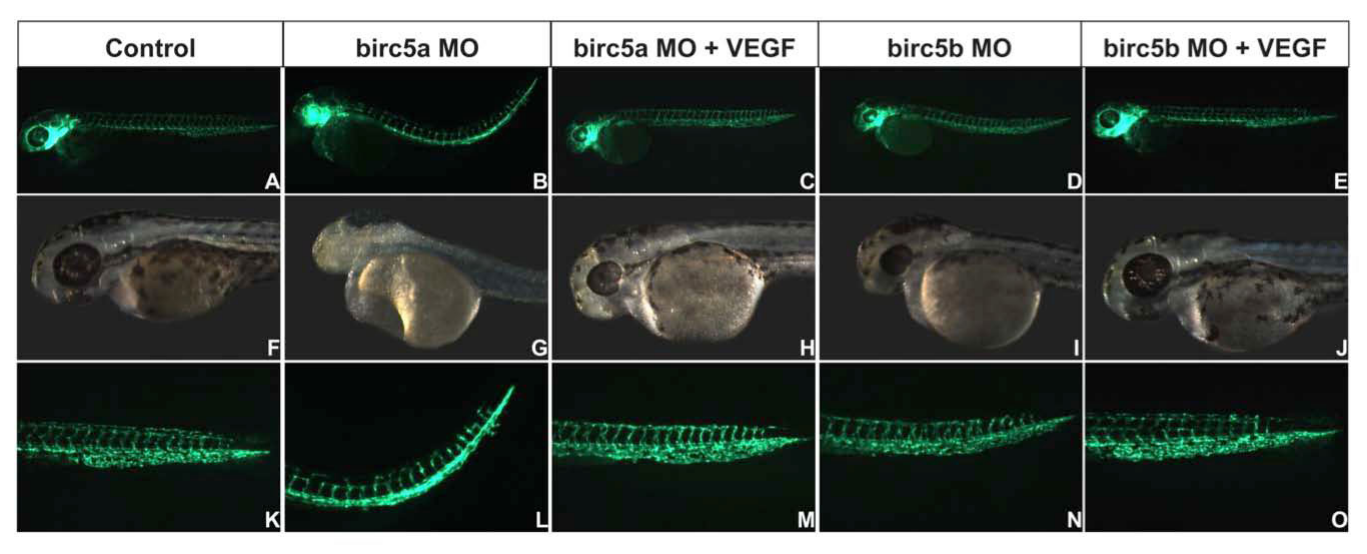
**Rescue of *Birc5 *knockdown phenotypes with VEGF mRNA**. *Birc5a *or *Birc5b *was depleted with 2 ng or 4 ng, respectively, of corresponding morpholino into Tg(*Fli*:eGFP) embryos, alone or with human VEGF mRNA. Embryos were evaluated at 2 dpf. Morpholino-induced angiogenic, cardiac and neurodevelopmental defects were reversed by VEGF.

Vascular endothelial growth factor (VEGF) has vasculo-angiogenic, neurogenic, cardiogenic and hematopoietic properties, the effects on endothelial cells mediated in part by upregulating survivin [37]. Ma et al [16] demonstrated that VEGF protein upregulates expression of *Birc5a *in zebrafish at 96 hpf. We evaluated whether VEGF could rescue the phenotypes induced by depletion of the 2 zebrafish *survivin *genes. Human VEGF mRNA (500 pg) or vehicle was injected into *Tg(Fli:EGFP) *embryos alone, or with a control morpholino, or with maximum dose morpholinos to deplete either *Birc5a *or *Birc5b *(Figure 8). Embryos were evaluated at 2 dpf. In conjunction with *Birc5a *morpholino knockdowns (n = 86), VEGF mRNA reduced the incidence of neural and vascular defects to 43% and 37%, respectively, of that observed with *Birc5a *morpholino alone. VEGF also rescued the phenotype induced by *Birc5b *depletion. Thus, administration of VEGF mRNA (n = 85) with *Birc5b *morpholino decreased the incidence of vascular and neural defects in *Birc5b *morphants (n = 110), to 20% and 24%, respectively, of that found with *Birc5b *morpholino alone. The results demonstrate that VEGF may protect the integrity of the neural and vascular systems from single *birc5 *gene depletion, possibly via a compensatory increase in expression of the second *birc5 *gene.

## Discussion and conclusion

In attempting to elucidate the physiologic relevance of the inhibitor of apoptosis protein, survivin, we have utilized the zebrafish model and characterized the expression patterns and functions of its two genes in early development. Our studies extend those of Ma et al [16], who first reported that *Birc5a *has angiogenic, but not vasculogenic, properties, a discrepancy that may be partly explained by the fact that their studies were restricted to developmental time points no earlier than 22 hpf. Several novel insights are provided by our work. Both *Birc5a *and *Birc5b *are expressed predominantly by neural, vascular, and ocular structures, and in the somites/somite boundaries; both inhibit apoptosis and promote cell proliferation; and both contribute to normal vasculogenesis, angiogenesis, cardiogenesis, neurogenesis and hematopoiesis. No other member of the IAP family has been shown to have similarly profound developmental effects on multiple organ systems following gene inactivation or knockdown in small animal models [38].

Our findings are consistent with immunohistochemical analyses and *survivin *gene inactivation studies in the mouse embryo, where the single *survivin *gene is essential for survival, and also plays a key role in angiogenesis, neurogenesis, cardiogenesis and hematopoiesis [5,11,13,14]. Our studies therefore support the concept that the zebrafish paralogs, *Birc5a *and *Birc5b*, largely recapitulate the properties of the ancestral gene, represented in the mouse, thereby rationalizing the use of this model to elucidate the role of survivin in health and disease in higher animals.

While the two zebrafish *survivin *genes have indistinguishable patterns of expression during development, in all of our assays, except those for hematopoiesis, depletion of *Birc5a *resulted in more severe, and sometimes distinct phenotypes. For example, only *Birc5a *depletion caused marked alterations in ISV patterning. This phenotype would be most readily attributed to expression of *Birc5a *at the somite boundaries, where VEGF [39] and other guidance molecules play a crucial role in vessel patterning (reviewed in [25]). However, *Birc5b *is also expressed in the somites/somite boundaries, and its depletion had no effect on the ISVs. Thus, further studies, including analyses of the expression profiles of relevant guidance molecules, will be required to further elucidate the specific properties of each *Birc5 *gene in the somites.

Functional differences between the two *Birc5 *genes were additionally uncovered by titering the respective morpholino doses to evaluate effects of each paralog on apoptosis and cell proliferation in the neural tube and vascular structures. By this approach, *Birc5a *was found to be more effective at protecting the neural structures, while *Birc5b *was more effective at protecting the caudal vein plexus. The latter is interesting, because the caudal vein plexus is a major site for primitive hematopoiesis, a process that was more prominently disturbed by *Birc5b *depletion. Nonetheless, administration of either mRNA could effectively rescue the phenotypes induced by depletion of the other. Thus, the physiologic relevance of the functional differences between the two *survivin *paralogs is not fully delineated, and it appears that each may act to compensate for deficiencies of the other.

A common feature of both *Birc5 *genes, not previously recognized, is that their expression in the somites and axial vessels is transient and restricted to an early developmental time period, both essentially gone by 3 dpf. Up until that time, the axial vessels and the ISVs are generated, and based on the morpholino knockdowns, survivin is critical for formation of these structures, after which survivin is no longer necessary at those sites. Interestingly, the IAP *Birc2 *is expressed in the vasculature of zebrafish beginning at 54 hpf, whereupon it is required to maintain endothelial cell integrity [40]. One could speculate that there exists an intrinsic molecular switch that is "flipped" at 2–3 dpf, when *Birc5 *expression turns off, and *Birc2 *turns on, the latter which is required to form a more complex vascular network. Characterization of such a switch mechanism could enhance our understanding of the regulation of angiogenesis.

In mice depleted of endothelial survivin, embryonic heart development was abnormal, and the mutant endocardial lineage cells could not support epithelial-mesenchymal transformation (EMT) [14]. In both *survivin *gene knockdowns in the zebrafish, we also observed abnormalities in cardiogenesis – more prominent with *Birc5a *depletion. Fate-mapping studies in zebrafish have revealed that formation of the atrium, ventricle and a-v valves depends on the integrity of the cardiac neural crest cells [36], while intracardiac fluid forces also contribute to normal heart development [33]. Thus, the etiology of the abnormalities in cardiogenesis in the *Birc5 *morphants may be multifactorial, i.e. secondary to the circulation defect and/or due to loss of the cardiac neural crest cells. Further study is required to elucidate the relevant *Birc5*-dependent pro-survival pathways for these neural crest cells. Nonetheless, the zebrafish and mouse models highlight the importance of survivin in heart development. Just as single-nucleotide polymorphisms of the *VEGF *gene have been linked to congenital valvuloseptal defects [41], the possibility that functional alterations in survivin expression might underlie congenital heart defects is worthy of consideration.

The prominent role that survivin plays in regulating vasculo-angiogenesis, neurogenesis, cardiogenesis and hematopoiesis supports the widely accepted notion of co-ordinately regulated development of these systems (reviewed in [42]). For example, the Eph/ephrins regulate fasciculation and guidance of axons, direct neural crest cell fate and migration, modulate neural progenitor cell survival, while also being important for cardiovascular development [43] and erythropoiesis [44]. Vascular endothelial growth factor (VEGF), although best characterized as a critical mediator of vasculogenesis and angiogenesis, also has direct effects on the nervous system (reviewed in [45]). VEGF protects neural cells from hypoxia, facilitates axonal outgrowth, promotes endothelial release of neurogenic factors [46], and induces neural stem cell proliferation [47]. VEGF also promotes hematopoiesis [48], and altered regulation of VEGF results in profound defects in heart development [49]. These properties of VEGF are confirmed in our studies, where the neural and cardiovascular phenotypes induced by depletion of *Birc5a *or *Birc5b*, were rescued by VEGF. Although not tested, it is likely that upon depletion of one *Birc5 *gene, the exogenous VEGF upregulated expression of the other, which in turn compensated for the phenotypic defects. In that respect, Ma et al [37] indeed, demonstrated that VEGF protein can increase accumulation of *Birc5a *mRNA in zebrafish embryos. Beyond the zebrafish model system, upregulation of survivin in endothelial cells has been well-documented [37]. The finding that survivin is also a downstream effector of VEGF in the neurologic system, and that *survivin *transcripts are highly expressed in neural progenitor cells [50], implies that the *survivin *gene in humans may, similar to VEGF [51], be a modifier in the progression of neural diseases, such as amyotrophic lateral sclerosis, and thus have the potential as a therapeutic target.

By regulating cell proliferation and apoptosis, the two zebrafish *survivin *genes, *Birc5a *and *Birc5b*, play important roles in multiple biologic systems. Although we have not examined all organs during development; nor have we yet evaluated its role in the eye; the prominent effects of *Birc5 *gene depletion on the cardiac, neurovascular and hematopoietic systems in the zebrafish embryo suggest that survivin has temporal and tissue-specific properties. In view of the apparent functional overlap with the murine and human survivin orthologues, the zebrafish model provides an exceptional opportunity to examine the physiologic relevance of the complex molecular and biochemical pathways that govern cell survival and apoptosis. The insights gained will lead to the development of safer, targeted therapeutics for a spectrum of cardio-vascular, hematopoeitic and neurologic diseases, and a better understanding of the etiology and genetics of an array of congenital diseases.

## Methods

### Zebrafish strains and maintenance

Wild-type AB zebrafish and *Tg*(*Flk1:EGFP*), *Tg*(*Fli*:EGFP)*y1*, and *Tg(gata1:GFP) *zebrafish were maintained under standard laboratory conditions [22]. Embryos were grown in 0.003% 1-phenyl-2-thiourea (PTU, Sigma, Bornem, Belgium) in 0.3× Danieau (17 mM NaCl, 0.21 mM KCl, 0.12 mM MgSO_4_.7H_2_O, 0.18 mMCa(NO_3_)_2_, 1.5 mM Hepes (Fluka, Bornem, Belgium) pH 7.6) starting from 24 hpf, were kept at 28.5°C, and staged according to standard criteria [52]. After 24 hours, the chorion was removed with trypsin 1.5 mg/ml (Sigma). All animal studies were approved by the University of Leuven Animal Studies Ethics Commission.

### Digoxigenin-labeled RNA probes for *in situ *hybridization

cDNAs were cloned into the pGEM-T Easy vector (Promega, Leiden, the Netherlands). For detection of *Birc5a*, primer pairs: Birc5a-F1/R1, Birc5a-F2/R2 and Birc5a-F3/R3 (see Additional file 2) were used to generate amplicons, respectively, of 902 bp (3' coding region + UTR), 344 bp (only 3' UTR) and 429 bp (coding region). For detection of *Birc5b*, probes were made with the following primer pairs: Birc5b-F1/R1 and Birc5b-F2/R2, generating amplicons, respectively, of 491 bp (coding region + 3' UTR) and 387 bp (coding region). After linearization with Spe1 and Nco1, antisense riboprobes were prepared with the DIG RNA labeling kit (Roche, Vilvoorde, Belgium) according to the manufacturer's instructions and purified with the RNeasy mini kit (Qiagen, Venlo, the Netherlands).

### Flow cytometry

*Tg(Gata1:GFP) *zebrafish embryos were injected at the 1-cell stage, and at 14 hpf, they were dechorionated and digested with 0.05% trypsin/EDTA for 15 minutes at 28°C. A single cell suspension was obtained by pipetting up and down, after which 100% fetal calf serum (FCS) was added. Cells were filtered through a 40 μM cell strainer, washed with 2% FCS/PBS, and the percentage of GFP-positive cells was measured by flow cytometry (FacsCalibur, BD Biosciences).

### mRNA preparation

cDNAs in plasmid vectors were linearized with the appropriate restriction enzyme, and the digested DNA was ethanol precipitated. mRNA for each *survivin *gene transcript or *vegf *was then prepared according to the manufacturers instructions (Ambion, Lennik, Belgium).

### Real-time PCR

Quantitative real-time PCR was performed to examine the expression of different hematopoietic genes using SYBR green (Applied Biosystems, Belgium) according to manufacturers instructions. The sequence of the oligonucleotide primers used are as reported by Ma et al [29]. The β-actin mRNA levels were used as an internal reference.

### Morpholino and mRNA injection

Morpholinos (Gene Tools LLC, Oregon, USA) were targeted against the ATG or 5'UTR of Birc5a and Birc5b. Morpholinos and synthetic mRNAs were diluted in 1.5% phenol red (Sigma) in 0.2 M KC. Fertilized zebrafish eggs at the 1–4 cell stage were positioned into agar slots, and using a Femtojet (Eppendorf, Haasrode, Belgium), a micromanipulator (Narishige, New York, USA) and a Zeiss-stemi 2000-C light microscope (Zeiss, Zaventum, Belgium), eggs were injected with 1 nl of morpholino using back-filled fine borosilicate, glass capillary needles.

### Detection of apoptosis and cell proliferation

Embryos were fixed in 4% paraformaldehyde overnight at 4°C and stored in methanol at -20°C until further processing. *In situ *hybridization was performed as described [17]. For apoptosis and cell proliferation studies, embryos were first rehydrated with decreasing concentrations of methanol, washed with PBST (PBS, 0.1% Tween 20), treated with proteinase K (10 mg/ml) at 37°C for 30 to 60 minutes, refixed with 4% paraformaldehyde, washed in PBST, and lastly incubated with 0.1 M citrate solution (0.1% citrate/PBS/Triton). TUNEL staining was performed with the ApopTag kit (Chemicon, Heule, Belgium). Cell proliferation was visualized using antibodies against phosphorylated phospho-histone H3 (Upstate, Huissen, the Netherlands). After mounting in 3% methylcellulose (Sigma) in PBS, embryos were visualized by light microscopy or with a Zeiss Lumar fluorescence stereomicroscope, and pictures were taken under green fluorescence *Tg*(*Fli:eGFP*) and red fluorescence (apoptotic cells or proliferating cells) with a Zeiss AxioCam MRc digital camera. For each embryo, the entire caudal vein plexus and corresponding overlying neural tube was delineated, and apoptotic cells or proliferating cells were manually counted. 30 embryos were analyzed per condition and 3 experiments were performed. Data were compared with a standard Student t-test.

## Authors' contributions

MD and FZ carried out *in situ *hybridizations and microscopy, and designed experiments, while MD also drafted the manuscript. AD, IB and MM prepared all riboprobes, did cDNA cloning and sequencing, and helped with *in situ *hybridizations, acquisition of data and analyses. MA provided continuous intellectual input, evaluation and interpretation of data, and writing of the manuscript. EC conceived, designed and co-ordinated the project, and drafted the manuscript. All authors read and approved the final manuscript.

## Supplementary Material

Additional file 1 **A**. **Using the CLUSTAL W program, protein sequences of human, mouse, Xenopus and zebrafish survivin were aligned.B**. The overall similarity (in %) at the level of amino acid sequence between survivins of human, mouse, Xenopus and zebrafish were compared. Homo, *Homo sapiens*; Mus, *Mus musculus*; XT, *Xenopus tropicalis*; Birc5, *danio rerio*; XL, *Xenopus laevis*; * identical amino acid; conserved change; highly conserved change. Click here for file

Additional file 2 **Primers used to generate *in situ *hybridization probes.** Click here for file

Additional file 3 **Morpholinos used for the knockdown of *Birc5a *and *Birc5b*.** Click here for file

Additional file 4 **An oligonucleotide for each zebrafish *survivin*gene containing the binding sites of the different morpholinos directed against the 5' UTR and the ATG of each gene, was cloned into the pCAG-T7-luciferase plasmid, resulting in the pCAG-T7-luciferase-Birc5a and pCAG-T7-luciferase-Birc5b plasmids.** These plasmids, together with varying doses of *Birc5 *morpholinos, were used in an *in vitro*transcription/translation assay, as described [18]. Experiments were performed in triplicate. Luciferase activity was measured in arbitrary light units that are noted in the Y-axis. *Birc5a *is only depleted by the *Birc5a *morpholino (B-F) and not by the *Birc5b *morpholino (G-K). Similarly, *Birc5b *is only depleted by the *Birc5b *morpholino (R-V) and not by the *Birc5a *morpholino (M-Q). *Birc5a *plasmid: pCAG-T7-luciferase-Birc5a; *Birc5b *plasmid: pCAG-T7-luciferase-Birc5b; MO1: *Birc5a *morpholino; MO2: *Birc5b *morpholino. Click here for file

Additional file 5 **Confocal microscopy was used to visualize a cross sectional view from the of top of the head region of a 3 dpf zebrafish embryo, revealing the different vascular structures.** Click here for file

Additional file 6 **When compared to the control (Movie S1), depletion of *Birc5a *with 2 ng of morpholino results in striking underdevelopment and irregular patterning of several blood vessels in the head of the embryo.** Click here for file

Additional file 7 **When compared to the control (Movie S1), knockdown of *Birc5b *with 4 ng of morpholino leads to underdevelopment of vascular structures in the head, although to a lesser extent than in the *Birc5a *morphants (Movie S2).** Click here for file
