# Supplementary figures and images for: Role of the 2 zebrafish survivin genes in vasculo-angiogenesis, neurogenesis, cardiogenesis and hematopoiesis
Source: BMC Dev Biol. 2009 Mar 26;9:25. doi: 10.1186/1471-213X-9-25 (PMC2670274; doi:10.1186/1471-213X-9-25)

## Slide 1
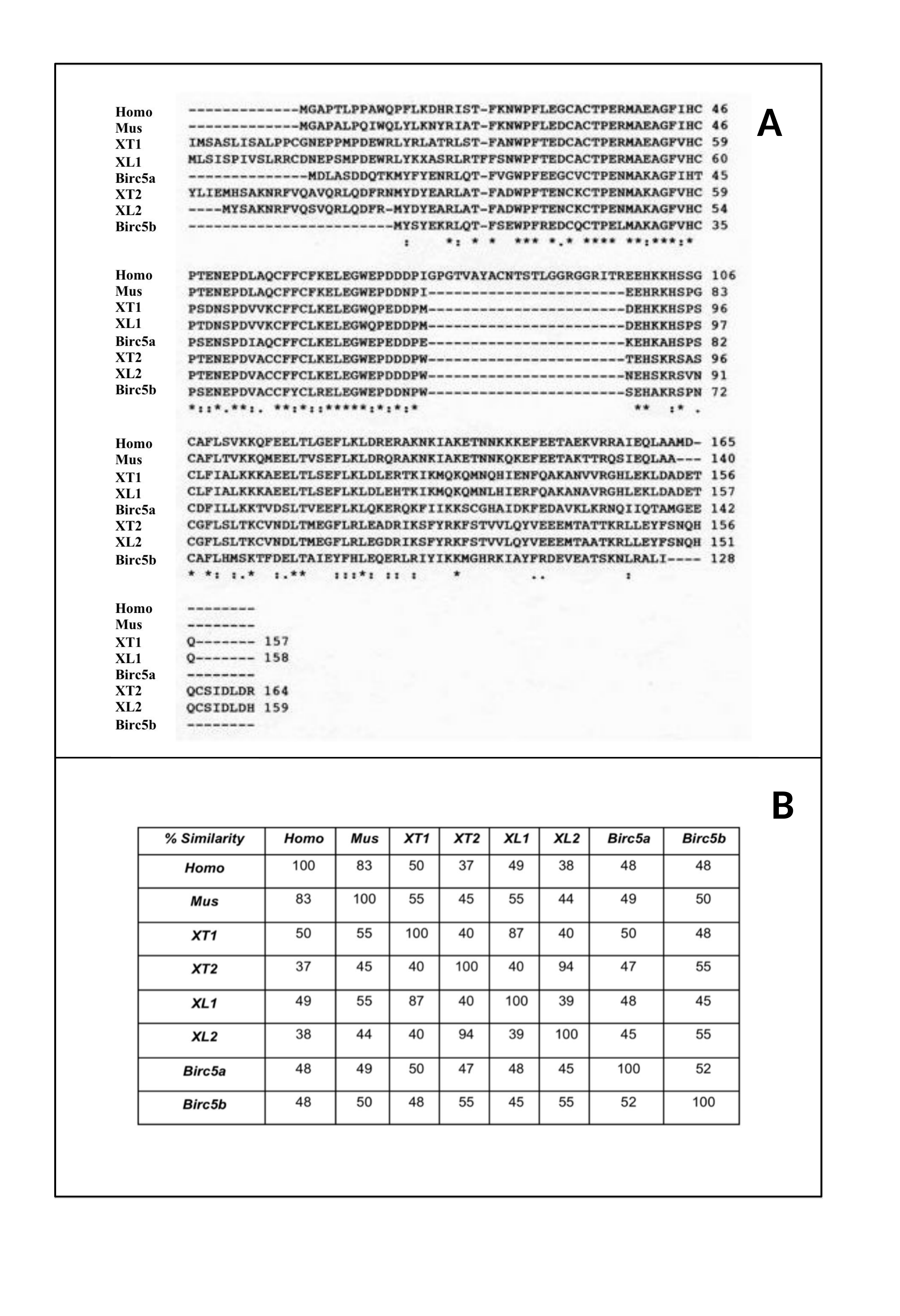

A
B

Supplement: Additional file 1 — A. Using the CLUSTAL W program, protein sequences of human, mouse, Xenopus and zebrafish survivin were aligned.B. The overall similarity (in %) at the level of amino acid sequence between survivins of human, mouse, Xenopus and zebrafish were compared. Homo, Homo sapiens; Mus, Mus musculus; XT, Xenopus tropicalis; Birc5, danio rerio; XL, Xenopus laevis; * identical amino acid; conserved change; highly conserved change. [file 1471-213X-9-25-S1.ppt]
