# Supplementary material for: Role of the 2 zebrafish survivin genes in vasculo-angiogenesis, neurogenesis, cardiogenesis and hematopoiesis
Source: BMC Dev Biol. 2009 Mar 26;9:25. doi: 10.1186/1471-213X-9-25 (PMC2670274; doi:10.1186/1471-213X-9-25)
Supplement: Additional file 2 — Primers used to generate in situ hybridization probes. [file 1471-213X-9-25-S2.rtf]

	Primers used to generate in situ hybridization probes	
Birc5a-F1	TCTGTCTGAAAGAGCTGGAA	
Birc5a-R1	TTATCAACAAATAGTACAA	
Birc5a-F2	AGAGGTTCATTGAGAGCAT	
Birc5a-R2	CAGGAGATGCACAATTGT	
Birc5a-F3	GAATTCTGGATCTTGCAATGAT	
Birc5a-R3	GAATTCTCATTCCTCTCCCATCG	
Birc5b-F1	CCTCCAGACTGCTGTGTCCTTT	
Birc5b-R1	AAACAAACATTTAATTTATTG	
Birc5b F2	GAATTCATGTATAGTTATGAAAAAAG	
Birc5b-R2	AGAATTCTCAAATAAGAGCTCTCAAA	
