# Supplementary material for: Role of the 2 zebrafish survivin genes in vasculo-angiogenesis, neurogenesis, cardiogenesis and hematopoiesis
Source: BMC Dev Biol. 2009 Mar 26;9:25. doi: 10.1186/1471-213X-9-25 (PMC2670274; doi:10.1186/1471-213X-9-25)
Supplement: Additional file 3 — Morpholinos used for the knockdown of Birc5a and Birc5b. [file 1471-213X-9-25-S3.rtf]

	Morpholinos used for the knockdown of Birc5a and Birc5b	
Birc5a -ATG	TGCAAGATCCATTTTGTGGGAGGTT	
Birc5a -UTR	GTGTGGAAATTAAACAAAAGACAAC	
Birc5a -Splice 	AAGACACGGACTCACTCAGGGTCAT	
Birc5b-ATG	GAAGTCTTTTTTCATAACTATACAT	
Birc5b-ATG2	ATCTGATTAAAGGACAGCAGTCTGG	
Birc5b-UTR	CTGATTAAAGGACAGCAGTCTGGAG	
Birc5b-Splice	CGCGCTTTCCACTCACCAGTTCTGG	
Control	CCTCTTACCTCAGTTACAATTTATA	
