# Supplementary material for: Role of the 2 zebrafish survivin genes in vasculo-angiogenesis, neurogenesis, cardiogenesis and hematopoiesis
Source: BMC Dev Biol. 2009 Mar 26;9:25. doi: 10.1186/1471-213X-9-25 (PMC2670274; doi:10.1186/1471-213X-9-25)
Supplement: Additional file 4 — An oligonucleotide for each zebrafish survivingene containing the binding sites of the different morpholinos directed against the 5' UTR and the ATG of each gene, was cloned into the pCAG-T7-luciferase plasmid, resulting in the pCAG-T7-luciferase-Birc5a and pCAG-T7-luciferase-Birc5b plasmids. These plasmids, together with varying doses of Birc5 morpholinos, were used in an in vitrotranscription/translation assay, as described [18]. Experiments were performed in triplicate. Luciferase activity was measured in arbitrary light units that are noted in the Y-axis. Birc5a is only depleted by the Birc5a morpholino (B-F) and not by the Birc5b morpholino (G-K). Similarly, Birc5b is only depleted by the Birc5b morpholino (R-V) and not by the Birc5a morpholino (M-Q). Birc5a plasmid: pCAG-T7-luciferase-Birc5a; Birc5b plasmid: pCAG-T7-luciferase-Birc5b; MO1: Birc5a morpholino; MO2: Birc5b morpholino. [file 1471-213X-9-25-S4.ppt]

## Slide 1
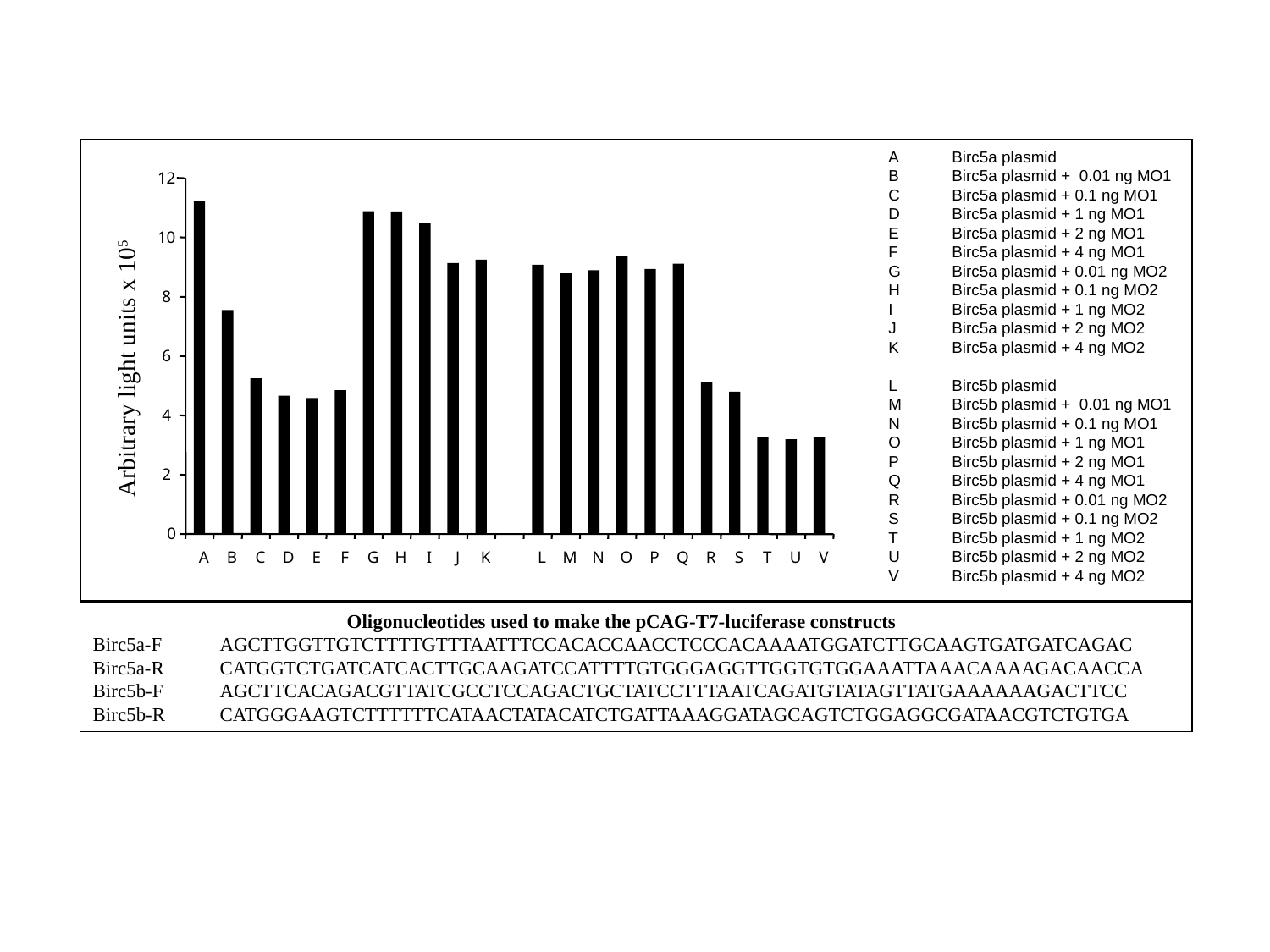

A	Birc5a plasmid
B	Birc5a plasmid + 0.01 ng MO1
C	Birc5a plasmid + 0.1 ng MO1
D	Birc5a plasmid + 1 ng MO1
E	Birc5a plasmid + 2 ng MO1
F	Birc5a plasmid + 4 ng MO1
G	Birc5a plasmid + 0.01 ng MO2
H	Birc5a plasmid + 0.1 ng MO2
I	Birc5a plasmid + 1 ng MO2
J	Birc5a plasmid + 2 ng MO2
K	Birc5a plasmid + 4 ng MO2
L	Birc5b plasmid
M	Birc5b plasmid + 0.01 ng MO1
N	Birc5b plasmid + 0.1 ng MO1
O	Birc5b plasmid + 1 ng MO1
P	Birc5b plasmid + 2 ng MO1
Q	Birc5b plasmid + 4 ng MO1
R	Birc5b plasmid + 0.01 ng MO2
S	Birc5b plasmid + 0.1 ng MO2
T	Birc5b plasmid + 1 ng MO2
U	Birc5b plasmid + 2 ng MO2
V	Birc5b plasmid + 4 ng MO2
12
10
8
6
Arbitrary light units x 105
4
2
0
A
B
C
D
E
F
G
H
I
J
K
L
M
N
O
P
Q
R
S
T
U
V
				Oligonucleotides used to make the pCAG-T7-luciferase constructs
Birc5a-F	AGCTTGGTTGTCTTTTGTTTAATTTCCACACCAACCTCCCACAAAATGGATCTTGCAAGTGATGATCAGAC
Birc5a-R	CATGGTCTGATCATCACTTGCAAGATCCATTTTGTGGGAGGTTGGTGTGGAAATTAAACAAAAGACAACCA
Birc5b-F	AGCTTCACAGACGTTATCGCCTCCAGACTGCTATCCTTTAATCAGATGTATAGTTATGAAAAAAGACTTCC
Birc5b-R	CATGGGAAGTCTTTTTTCATAACTATACATCTGATTAAAGGATAGCAGTCTGGAGGCGATAACGTCTGTGA
